# CERN-based experiments and Monte-Carlo studies on focused dose delivery with very high energy electron (VHEE) beams for radiotherapy applications

**DOI:** 10.1038/s41598-024-60997-5

**Published:** 2024-05-15

**Authors:** L. Whitmore, R. I. Mackay, M. van Herk, P. Korysko, W. Farabolini, A. Malyzhenkov, R. Corsini, R. M. Jones

**Affiliations:** 1 Department of Physics and Astronomy, University of Manchester, Manchester, M13 9PL UK; 2 The Cockcroft Institute of Science and Technology, Daresbury, UK; 3 Present Address: Department of Radiation Physics, University of Texas MD Anderson Cancer Center, Houston, USA; 4 Division of Cancer Sciences, School of Medical Sciences, Faculty of Biology, Medicine and Health, The University of Manchester, Manchester, UK; 5 Christie Medical Physics and Engineering, The Christie NHS Foundation Trust, Manchester, UK; 6 Department of Physics, University of Oxford, Oxford, UK; 7 CERN, 1211 Geneva 23, Switzerland

**Keywords:** Cancer, Cancer therapy, Radiotherapy, Particle physics

## Abstract

Very High Energy Electron (VHEE) beams are a promising alternative to conventional radiotherapy due to their highly penetrating nature and their applicability as a modality for FLASH (ultra-high dose-rate) radiotherapy. The dose distributions due to VHEE need to be optimised; one option is through the use of quadrupole magnets to focus the beam, reducing the dose to healthy tissue and allowing for targeted dose delivery at conventional or FLASH dose-rates. This paper presents an in depth exploration of the focusing achievable at the current CLEAR (CERN Linear Electron Accelerator for Research) facility, for beam energies >200 MeV. A shorter, more optimal quadrupole setup was also investigated using the TOPAS code in Monte Carlo simulations, with dimensions and beam parameters more appropriate to a clinical situation. This work provides insight into how a focused VHEE radiotherapy beam delivery system might be achieved.

## Introduction

Radiotherapy aims to deliver a lethal dose to tumours whilst sparing healthy tissue as much as possible. Since its inception in the early 1900s, radiotherapy continues to be a speciality with rapidly changing technologies. Techniques such as intensity modulated radiotherapy (IMRT), volumetric arc therapy (VMAT) and image guided-radiotherapy (IGRT) have enabled highly conformal radiotherapy treatments to be delivered using photons^1–3^. The development of hospital-based proton centres, since Loma Linda in 1990^4^, has led to an expansion in proton radiotherapy. Protons have a beneficial dose deposition in the form of the Bragg peak, with high dose deposited at the distal beam end and minimal downstream dose. Proton radiotherapy is typically spot scanned; the delivery of narrow Bragg peaks of different energy are combined to produce a homogeneous Spread-Out Bragg Peak (SOBP) region. Proton beams are, however, highly sensitive to inhomogeneities such as air gaps^5^, and requiring highly accurate image guidance pre-treatment^6^. Heavy ion radiotherapy is also an emerging field, with a higher Relative Biological Effectiveness (RBE), though building the centres is significantly more expensive^7^ than the already expensive proton radiotherapy centres.

In recent years, interest has grown in using VHEE (Very High Energy Electrons, 50–250 MeV) for radiotherapy. This is due to the highly penetrating nature of VHEE beams, enabling deep-seated tumours to be reached, as well as the limited penumbral spreading (especially with higher beam energies)^8^, ease of magnetic steering, and insensitivity to inhomogeneities^9^. Treatment plans produced with VHEE have been shown to compete with or outperform photon VMAT plans^10^. Previous work has shown^11,12^ that focusing VHEE beams using quadrupole magnets improves the dose distribution by reducing the entrance dose, and allowing for the dose to be focused at a target location inside a water phantom.

VHEE has also been highlighted as a potential candidate for ultra-high dose-rate (FLASH) radiotherapy. Delivering radiotherapy at FLASH dose-rates (often quoted as average dose-rates > 40 Gy/s^13^) has been shown to produce similarly lethal doses to the tumour, with reduced toxic effects to healthy tissue compared to conventional radiotherapy^13–15^. Treatments with FLASH have mostly used low energy electrons (typically 6–25 MeV), though multiple centres are looking at other radiotherapy modalities to treat deeper-seated tumours^14,16^, including the FAST-01 clinical trial with protons^17^.

VHEE radiotherapy is not currently available clinically, but could potentially be treating patients within the next few years. Several facilities are currently proposed or under development. One such facility is FLASHDEEP^18^, which aims to be the first machine to delivery FLASH therapy to patients using VHEE beams. The accelerator will be an X-band linac with an accelerating gradient of approximately 100 MeV/m, and a footprint of approximately 10 m. FLASHDEEP aims to be starting clinical trials in 2025^19^. Another proposed facility is by the Lumitron/UC Irvine collaboration^20^, which aims to produce a VHEE X-band clinical machine with a beam energy of >100 MeV. Additionally, there is the proposed C-band linac at a VHEE-LINAC FLASH-RT Research Laboratory by Sapienza University^21^, which aims to produce a beam energy of approximately 100 MeV, which will be used initially for dosimetry and pre-clinical studies. Both the Lumitron and Sapienza designs aim to fit within a space similar to existing radiotherapy bunkers.

In addition to using RF technology to produce treatment electron beams, other groups aim to use plasma-wakefield acceleration as the source of VHEE beams with very high acceleration gradients^22,23^. Plasma wakefield acceleration potentially achieves >GeV/m^22,23^, but beam quality has been unsuitable for practical applications in medical facilities. However, recent work has indicated considerable improvements^24^, although this methodology is unlikely to be applicable to medical facilities within the next decade. In the meantime, experiments using VHEE beams are planned at VHEE test facilities including CLARA^25^ at Daresbury laboratory and the CLEAR facility at CERN^26^.

For widespread implementation of VHEE radiotherapy, future centres would ideally be capable of delivering highly conformal treatment plans, delivered at FLASH dose-rates. One potential method for delivering conformal FLASH VHEE therapy could be using quadrupole magnet focusing, which would reduce the entrance dose and allow for the dose to be targeted on the tumour. As FLASH radiotherapy is a rapidly developing field, the exact parameters for the FLASH effect to be observed have not yet been established. There is some research that implies that to achieve dose-rates across an entire treatment delivery, it may not be possible to use the techniques of spot-scanning, intensity modulated, or volume arc modulated beams delivered via multiple gantry angles, both of which require times on the order of seconds or minutes to produce. Focusing VHEE beams could potentially provide a method of conformality at FLASH dose-rates by providing another option which is faster to achieve than spot-scanning or multiple beam angles. The feasibility of this must investigated in future work by incorporating focused VHEE beams into a treatment planning system (TPS), though this is beyond the remit of this work.

This work presents experimentally focused VHEE beams obtained at the CLEAR (CERN Linear Electron Accelerator for Research)^26^ facility, which has a 70–220 MeV electron beam available for user experiments. It should be noted that the CLEAR facility is not optimised for VHEE or focused VHEE experiments, and has no plans to become a clinical or preclinical treatment centre. The work presented here shows focused VHEE beams produced in air and in a water phantom, demonstrating a straightforward method to change the focal point and therefore target location. In addition, a method for producing a uniform dose distribution is investigated for the first time, by inverting the final three quadrupole strengths. This work investigates methods for improving the focused dose further for clinical relevance using Monte Carlo simulations, reducing the size of the focusing system and exploring the effect of increasing the vacuum beampipe aperture for highly penetrating, focused VHEE in a water phantom.

## Results

**Figure 1:**
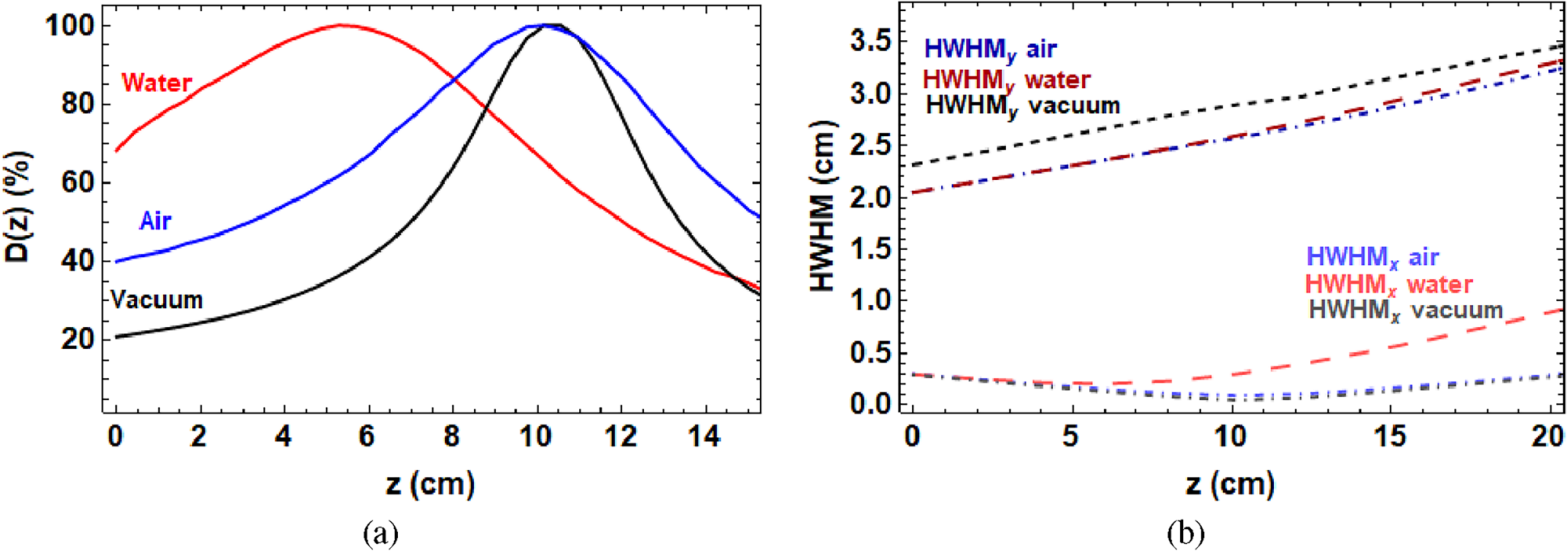
(**a**) TOPAS Monte Carlo simulations of the VHEE percentage on-axis dose in water, air and vacuum for the quadrupole currents shown in Supplementary Table 1 and 200 MeV beam energy. (**b**) Corresponding beam HWHM (Half Width Half Maxima) with respect to depth, showing the increased scattering in air and water, and the larger HWHM in vacuum due to the absence of collimation from the steel beampipe (of diameter 3.8 cm).

The method for focusing the VHEE beams is described in the “Methods” section. TOPAS Monte Carlo simulations^27^ comparing the resultant dose distributions for the same quadrupole strengths in air and water with the stainless steel beampipe, and also in vacuum without the stainless steel beampipe are shown in Fig. 1. The quadrupole currents used are shown in Supplementary Table 1, with the conversion to quadrupole strengths shown in Eq. (1). For a given set of quadrupole strengths within the CLEAR facility, as Fig. 1 indicates, focusing deeply in vacuum is the most straightforward to achieve. In water, focusing is less effective and at shallower depths.

Note that in Fig. 1 and all of the following results, the dose is recorded is the central axis dose.

**Figure 2:**
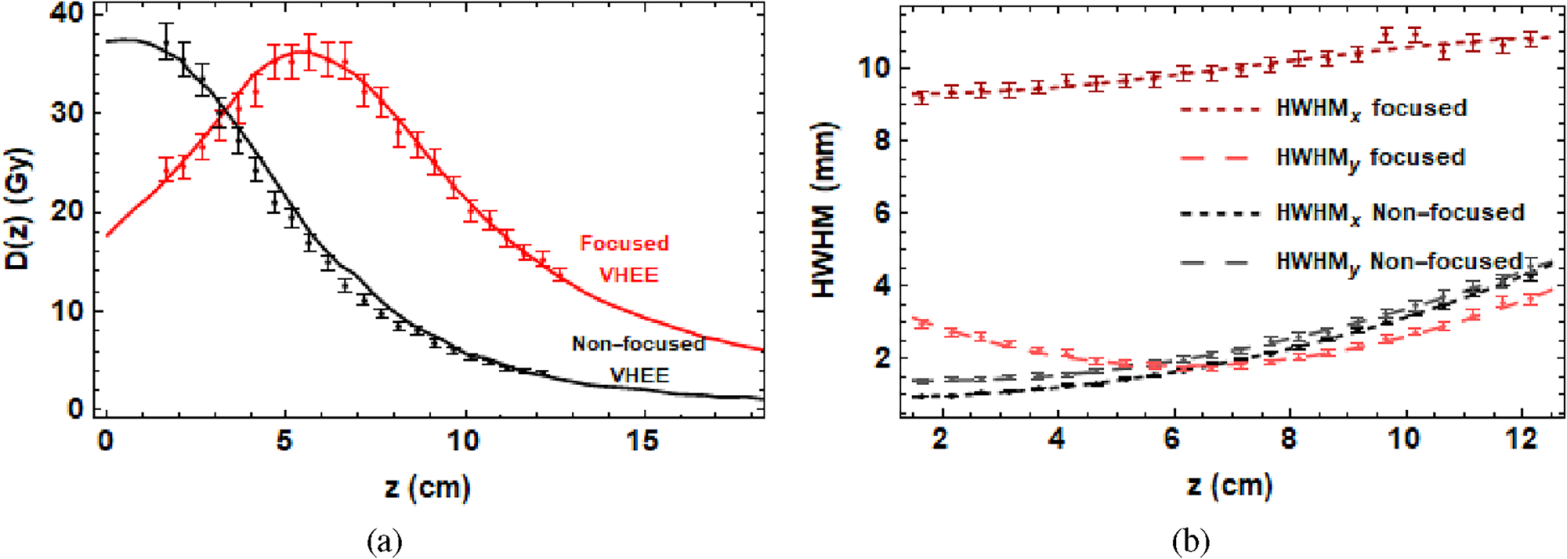
(**a**) VHEE percentage on-axis dose in water due to focused and non-focused electron beams. Points correspond to experimental data, and the lines result from Monte Carlo simulations. (**b**) The corresponding beam half-width-half-maximums (HWHMs) in the x-plane (focused) and the y-plane (diverging) as a function of depth.

At CLEAR, we found that the best focusing achievable in water was for a beam size that was wide and non-diverging in the x-plane, and small but strongly focused in the y-plane, resulting in a beam size decrease in the y-plane to approximately 50$\%$ at the focal point, shown in Fig. 2, for the quadrupole strengths shown in Supplementary Table 2 in the Supplementary Materials. Also shown in Fig. 2 is the result with no quadrupoles switched on, i.e. in the absence of focusing. The orientation of the beam is shown in Fig. 8. In all of the following results, an experimental beam energy of 201 MeV was used, with 2 MeV energy spread, with the same parameters used in the TOPAS Monte Carlo simulations.

The results in Fig. 2 are for the quadrupole currents shown in Supplementary Table 2. A YAG (yttrium aluminium garnet) screen was inserted at position 810 in Fig. 7 to artificially increase the beam emittance. The Twiss parameters $\alpha$, $\beta$ and the beam emittance, $\varepsilon$ (described in the Supplementary Materials) were then reconstructed at QFD760 using quadrupole scans performed using QDD870 and QF880 on another YAG screen. The reconstructed Twiss parameters used for the optimisations are shown in Supplementary Table 3. These Twiss parameters were then used to optimise focusing. The effects of changing the final quadrupole strength were tested experimentally at CLEAR first in air, shown in Supplementary Figs. 1 and 2 in the Supplementary Materials. These results were used as the basis for determining the quadrupole settings required to produce observable focused VHEE beams on the GafChromic films in water.

**Figure 3:**
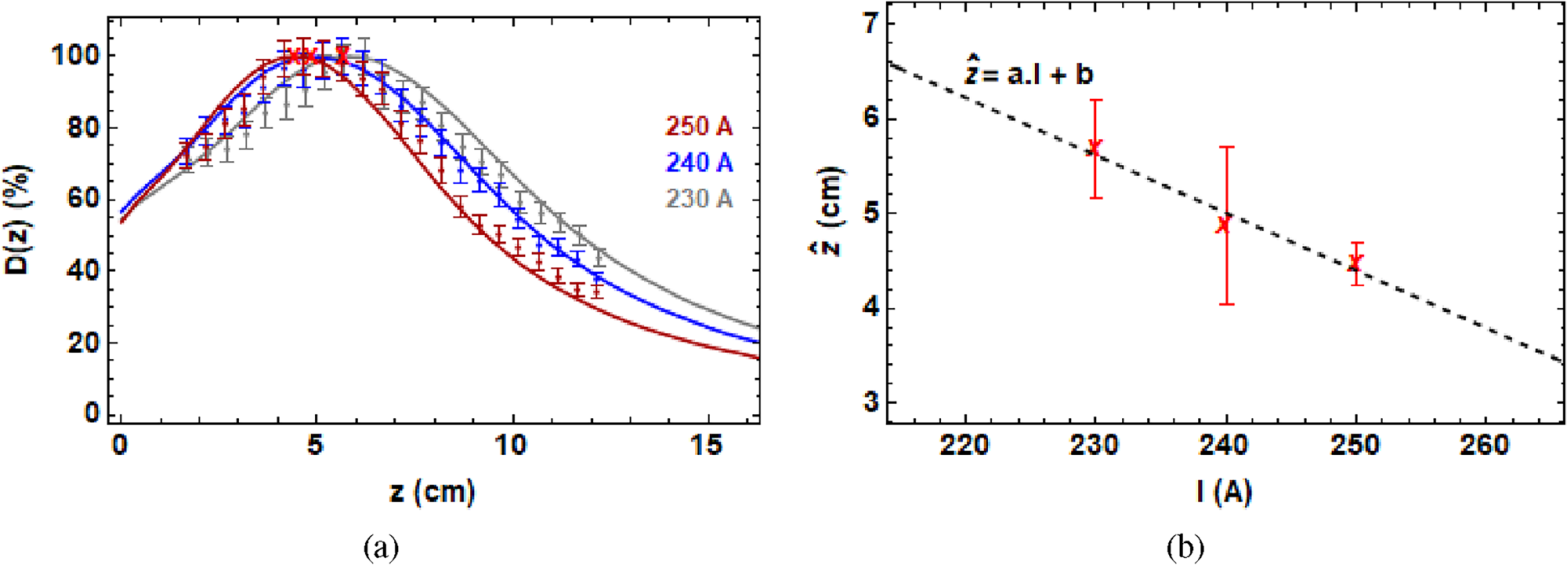
(**a**) Percentage on-axis dose due to changing the final quadrupole strength in water. Points are experimental data, and lines are Monte Carlo simulations. (**b**) A linear fit of peak dose position to final quadrupole current, $a=-0.06 \pm 0.01 \text { (cm/A)}$, $b=19.6\pm 2.8 \text { (cm)}$, adjusted $R^2$ = 0.999. The red crosses indicates the peaks in both (**a**) and (**b**).

It was found that due to the relatively narrow beampipe aperture and location of the quadrupoles, it was not possible to produce focused beams in water at depths greater than 6 cm with the fixed CLEAR beamline, which has not been optimised for focused VHEE beams, or radiotherapy applications. Previous simulation work predicted that changing the final quadrupole strength almost linearly changes the focal point in water^11^. The effect of changing the final quadrupole strength in water was investigated experimentally here for the first time and it is shown in Fig. 3.

The quadrupole currents used to produce the doses in Fig. 3 are the same as those shown in Supplementary Table 2, with the exception of the final quadrupole currents changed three times, to 230 A, 240 A and 250 A, and a beam energy of 201 MeV with 2 MeV energy spread.

These results show the focusing achievable at the CLEAR facility, which is not optimised for either focused VHEE or radiotherapy applications (and there are no plans for CLEAR to become a clinical or pre-clinical facility). They also demonstrate experimentally how the large beamline, small beampipe aperture and rigid positions of the quadrupoles impact the predicted focusing compared to Elegant^28^, which is an accelerator physics code that is used here to optimise for the focal strengths required to produce focused VHEE beams in a vacuum. The experimental result is also compared to the TOPAS Monte Carlo simulations.

**Figure 4:**
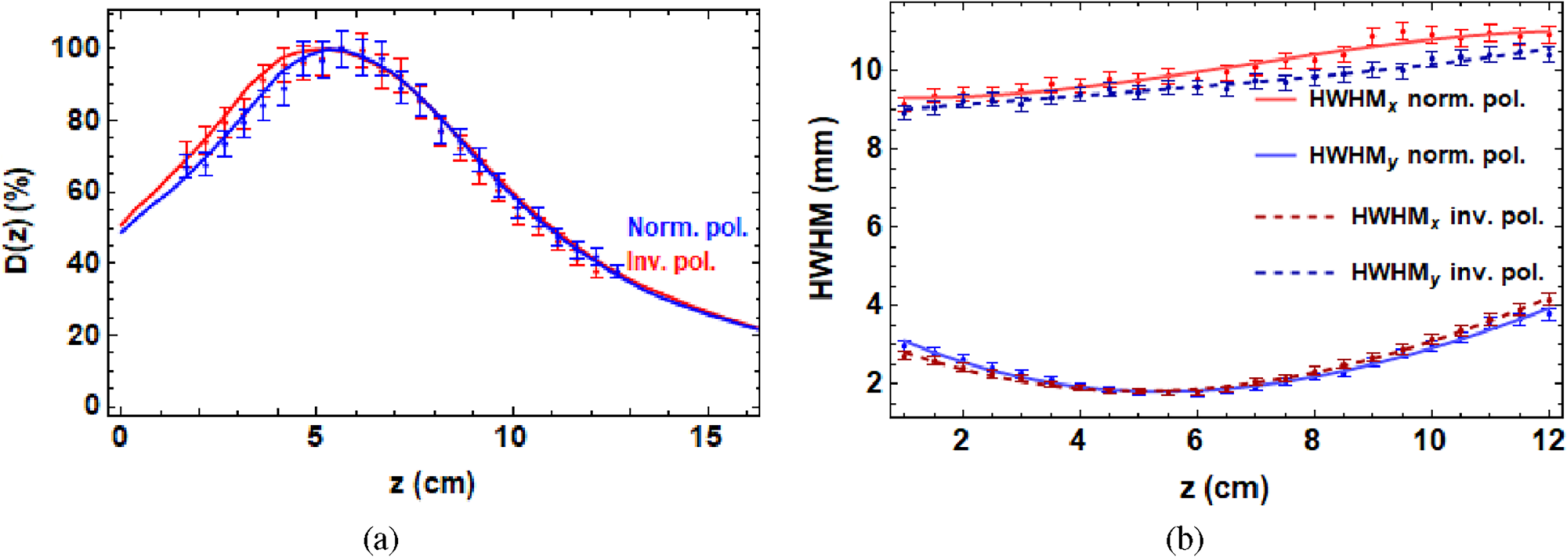
(**a**) On-axis dose in the water phantom at CLEAR due to normal (norm.) and inverted (inv.) polarity quadrupoles recorded on EBT-XD film (points) with the corresponding Monte Carlo simulations (lines). (**b**) The HWHMs with respect to depth recorded on EBT-XD films.

Finally, the effect of reversing the polarity of the final three quadrupoles was investigated experimentally at the CLEAR facility at CERN, with the results shown in Fig. 4.

The obtained on-axis dose distribution is almost symmetric and the beam size throughout the water phantom in the x-plane is reversed with that of the y-plane when the quadrupoles are inverted, and vice versa for the y-plane to x-plane. This method could be used to rapidly produce a symmetric dose distribution in 2D by combining the two dose distributions.

**Figure 5:**
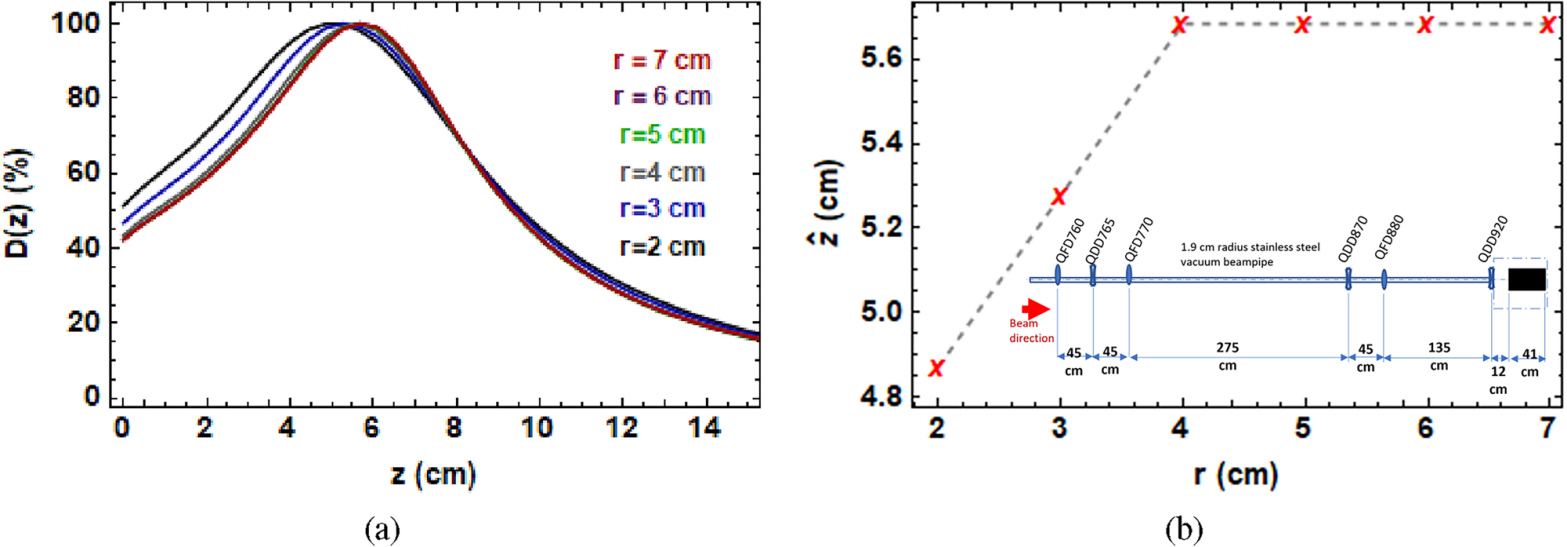
(**a**) Monte Carlo simulations of on-axis dose for the 240 A quadrupole settings stated previously, for different vacuum beampipe radii. (**b**) Position of maximum dose ($\hat{z}$) vs vacuum beampipe radius (*r*), showing plateau at *r* = 4 cm. Inset is the CLEAR beamline showing the large quadrupole spacings, particularly between QF3 and QD4, which is not optimal for producing focused VHEE beams.

For a more clinically relevant design with respect to the CLEAR beamline, specifically for focused VHEE radiotherapy, a larger beampipe aperture and more flexibility over quadrupole positions should be used. This will allow for a larger (ideally i.e. > 1 cm) beam size entering the phantom, it should be achievable to produce more tightly focused, deeply penetrating VHEE beams. To demonstrate the importance of beampipe aperture alone, the effect of increasing the beampipe radius for the current setup used at the CLEAR facility at CERN is shown in Monte Carlo simulations in Fig. 5.

This shows that simply increasing the vacuum beampipe radius from 2 to 4 cm would result in an improvement in focusing. This is because increasing the vacuum beampipe increases the penetration depth and reduces the entrance dose, producing sharper focused peaks than what were achieved with the smaller pipe. Increasing the beampipe further than this results in little to no improvement in focusing effect for this quadrupole configuration.

**Figure 6:**
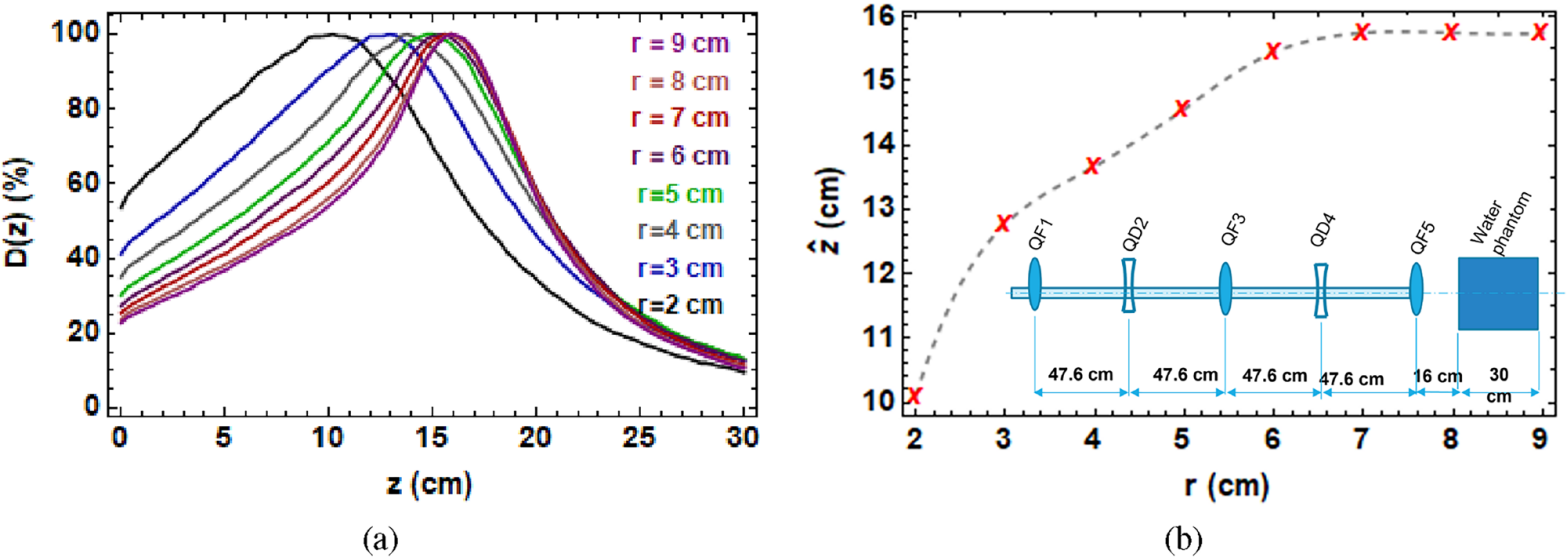
(**a**) Monte Carlo simulations of deeply penetrating, focused on-axis dose, for different vacuum beampipe radii. (**b**) Position of maximum dose ($\hat{z}$) in the water phantom vs vacuum beampipe radius. Inset is the optimised beamline showing the smaller, symmetric quadrupole spacings, which could potentially be useful clinically as it allows for both a more compact system and for focused beams at penetration depths of 15 cm or more within the water phantom.

In order to improve focusing further, allowing for clinically relevant focused beams with a more symmetric shape and focused to target deep-seated tumours at depths $\ge$ 10 cm, an improved quadrupole configuration would be required, which additionally should be more compact, to make it compatible with a clinical facility design. An example of a more compact focusing system, with five quadrupoles spaced with a 25 cm drift space between each quadrupole (see “Methods” section for details), is shown in Fig. 6, for a variety of vacuum beampipe sizes. The beam traverses the five quadrupoles inside the vacuum beampipe and then exits the final quadrupole into air and then a 30  cm $\times$ 30 cm $\times$ 30 cm water phantom, as shown in the schematic diagram in Fig. 10 in the “Methods” section.

This shows the effect of both the vacuum beampipe size, and the benefit of smaller spacings between the quadrupoles. Here, even with a small vacuum beam pipe size of 2 cm, it is possible to focus to depths of 10 cm; increasing the vacuum beam pipe to 5 cm increases this by a further 3 cm, and for sizes of 6 cm or greater, it is possible to target a depth of 15 cm with low entrance dose (20%), which is a significant improvement over no focusing, for a footprint which is smaller than a proton cyclotron facility. Further depths into the water phantom would be possible with a lower final quadrupole strength.

## Discussion

Previous work in focusing VHEE beams has mostly been simulation based^11,29^, with limited experimental studies to date^12^. This paper presents the first demonstration of multiple focused beams by changing only the final quadrupole strength, achieved at the CLEAR facility in CERN. Finally, it was shown that a symmetric distribution can be achieved by reversing the magnet polarities, albeit with the larger beam-width dominating the resulting symmetric distribution. Removing the restriction of fixed quadrupole magnet location, along with larger beam pipes, will allow better focussing in both planes, albeit with stronger focussing in one plane.

The focused VHEE beams are presented here to offer a method to deliver a targeted dose distribution rapidly, potentially without the need for spot scanning or gantry motion, which is assumed in most of the published VHEE treatment plans to date^10,30–32^. We do not expect the dose distributions with focused VHEE to outperform these, but rather that they offer an extra degree of freedom that could be used to deliver the dose distributions more rapidly, as may be required for FLASH treatment with electron beams. More careful studies are needed, but it is conceivable that FLASH effect may be reduced by spot scanning or mechanical gantry motion^33,34^.

A suite of beam optics simulations were performed in order to study the focusing effects illustrated herein. It was found that due to the experimental limitations of a fixed relatively narrow beampipe aperture (3.8 cm diameter), it was not possible to produce focused beams at depths deeper than approximately 6 cm in the water phantom. To achieve focused beams deeper in the water phantom would require an entrance beam size greater than the size of the current aperture of the beam pipe before entering the water phantom. The main reason for the need of a larger beam size is that it will facilitate larger focusing to overcome significant scattering that occurs in water. This limitation was less critical in air as the scattering effect is much less than in water, and so it was more straightforward to produce beams focussed more deeply into the phantom.

To be clear on the effect of the beneficial effects of increasing the beam pipe radius a study was performed. We discovered 4 cm would be sufficient to improve the focusing depth by a further 2 cm (to a depth of at least 8 cm). The practicality of this needs to be assessed using a detailed treatment planning system (TPS), comparing against the treatment plans produced with different beam energies. For the low penetration depths in this study, it is not foreseen that a full focusing system would be required, as VHEE beams with this energy already deliver a relatively flat dose profile^35^.

For focused VHEE beams anticipated to potentially treat deep-seated tumours, an improved quadrupole setup to that available at CLEAR would be required. However, there are at present no plans foreseen to treat patients at CLEAR as it remains a user facility for fundamental science experiments. Our study however, based on detailed Monte Carlo simulations, has revealed that delivery of focused VHEE beams for radiotherapy should be possible with a compact set of quadrupole magnets (five quadrupole magnets spaced from each by 25 cm should be sufficient). Wide bore quadrupole magnets and a large beampipe aperture will allow deeply penetrating, focused VHEE beams can reach targets of 15 cm or greater into patients. This is because focusing is a geometric effect meaning that for very strong focusing (and therefore, reduced entrance dose), a large beam at the exit of the final quadrupole with a strong focusing angle into the patient will be required. The usefulness of this compared to higher energy VHEE treatment plans must be assessed within a full treatment planning study. Focused VHEE beams could be a clinically relevant for the treatment of deep-seated tumours at FLASH dose-rates. It should be noted however that this would be less conformal solution than using a full VMAT or pencil beam spot-scanning style delivery.

The work shown here uses a water phantom. Future work should concentrate on using anthropomorphic phantoms, and incorporate the focused beams with treatment planning systems. Future experimental work on focused VHEE beams, either at CLEAR or a future VHEE facility (such as CLARA at Daresbury Laboratory^25^) with a larger vacuum beampipe aperture, will need modified quadrupole positions. The final three quadrupoles would need to be spaced as a triplet rather than the doublet, drift space, singlet combination presently at CLEAR, which limited the focusing depth achieved in this study. Future work will also include incorporating focused VHEE beams into a treatment planning system, to inform the development of a treatment centre capable of delivering conformal treatments targeting deep-seated tumours at FLASH dose-rates.

## Conclusions

This work demonstrates experimentally, for the first time, that changing the final quadrupole strength alone almost linearly changes the position of the dose delivered for VHEE beams at energies of 201 MeV, at the CLEAR facility at CERN. These results were shown both in air and in water, with shallower results in water due to the increased scattering effect and geometrically fixed beamline. Rotated dose distributions were achieved by inverting the polarity of the final three quadrupole magnets. In addition, the effect of the quadrupole aperture and spacings between quadrupoles were explored in Monte Carlo simulations, laying groundwork towards a future, focused VHEE radiotherapy centre by showing that with small spacings between quadrupoles (as little as 25 cm) and a large beampipe aperture, a focused VHEE delivery system could deliver target VHEE to depths of 15 cm or more, using a magnet system of less than 2 m total length. Focusing VHEE beams for radiotherapy could provide a convenient, rapid mechanism to deliver dose at FLASH dose-rates, with the requirement of a full incorporation of focused VHEE beams into a treatment planning system required as the next step.

## Methods

### CLEAR beamline

**Figure 7:**
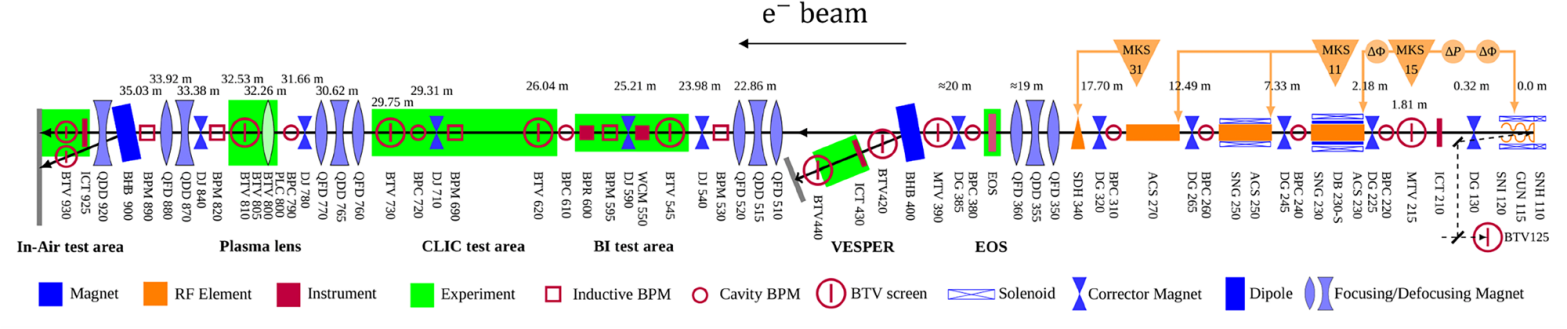
End-to-end diagram of the beamline at the CLEAR facility at CERN^26^. The water phantom, films and robot were installed at the in-air test stand, shown left-most, with the entrance of the water 12 cm after the exit of the final quadrupole. A vacuum beampipe runs from the start of the beamline to 139 cm after QFD880, after which a beam exit window is present and the beam continues to travel through air through the final quadrupole (QDD920) to the in air test stand, where the robot and water phantom are installed on a stage. The beam travels from right to left.

The experiment was conducted at the CLEAR beam line at CERN. A schematic of the experimental layout is shown in Fig. 7.

The CLEAR facility provides an electron beam covering a large range of parameters listed in Table 1. The electron beam is produced from a Cs_2_Te photocathode and is accelerated between 30 MeV and 220 MeV in a 20 m long linear accelerator. It composed of one RF (radio-frequency) photoinjector and three S-band accelerating structures, powered by two independent 3 GHz klystrons, followed by a 20m long experimental beam line. The full details of the beam parameters available at CLEAR are detailed in^36^.

**Table 1:** Updated list of CLEAR beam parameters.

Parameter	Value
Beam energy	30–220 MeV
Beam energy spread	$< 0.2 \%$ rms ($< 1$ MeV FWHM)
Bunch length RMS	0.1–10 ps
Bunch frequency	1.5 or 3.0 GHz
Bunch charge	0.005–1.6 nC
Norm. emittance	1–20 $\upmu$m
Bunches per pulse	1–200
Max. pulse charge	87 nC
Repetition rate	0.8333–10 Hz

S-band accelerating structures are those most commonly used in clinical linacs, but it should be noted that they have typical accelerating gradients of $\approx$20 MeV/m^37^, and as such are not optimised for a clinical VHEE machine. Rather, an accelerating structure with higher gradients, such as a C-band (gradients $\approx$ 50 MeV/m^21^) or X-band linac (gradients $\approx$100 MeV/m^38^) would make clinical implementation of VHEE much less space-intensive. Additionally, with the use of an optimised beam transport system, the total length of the machine can be reasonably expected to be <10 m, with the possibility of fitting inside pre-existing radiotherapy bunkers.

Earlier work, by the present authors, has shown it is possible to sharpen the focus of VHEE beam by using higher energies^11^. This is due to the reduced scattering of the beam and a greater penetration depth for higher energy VHEE beams. An additional dependence of focusing on beam energy is due to the relationship $g=E[MeV]\times K[m^{-2}]/300$^39^, where *g* is the quadrupole strength in T/m, *E* is the beam energy in MeV and *K* is the quadrupole strength in units of $m^{-2}$ provided by the matrix formulation for the effect of a quadrupole on an electron beam (see Supplementary Materials for more information). This shows that higher quadrupole strengths are required for higher beam energies, and the quadrupoles used at CLEAR are nominally designed for use at a beam energy of 200 MeV.

**Figure 8:**
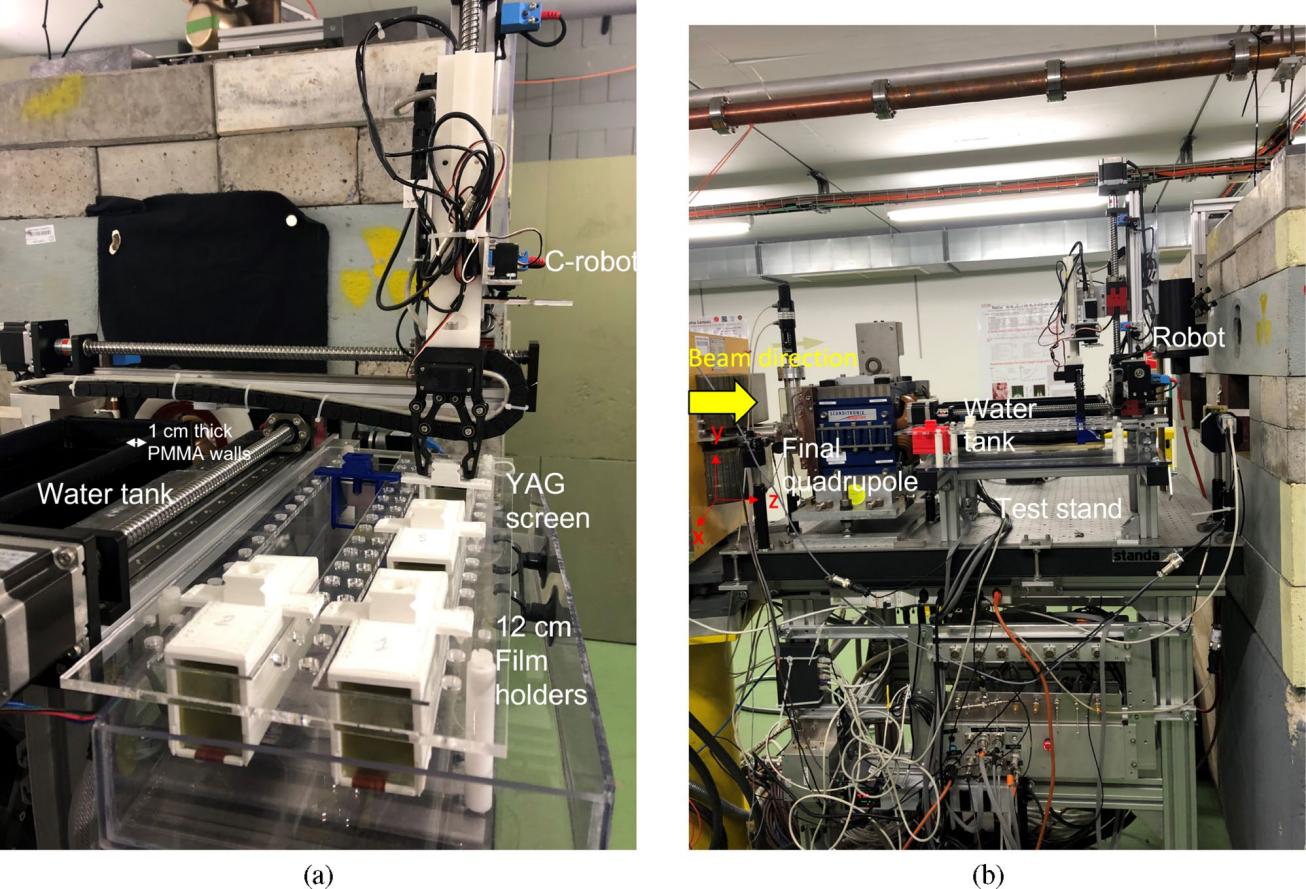
Shows the test stand area at the CLEAR facility. (**a**) Shows the water tank with 1 cm thick PMMA walls, the 12 cm long film holders and the C-shaped robot arm used to place the film holders in the plane of the beam. (**b**) Shows the test stand and final quadrupole, as well as the *x*-, *y*- and *z*-directions referred to throughout the manuscript.

In this experiment, a beam energy of 201 MeV was chosen, due to the optimal focusing achieved at this energy for the CLEAR beamline and quadrupole currents available. The electron source is a radio frequency (RF) gun followed by an S-band linac. The beam is focused through one quadrupole triplet, after which the beam is either directed to VESPER (The Very Energetic Electron facility for Space Planetary Exploration missions in harsh Radiative environments)^40^ test stand (see Fig. 7) where a dipole magnet is used to measure the beam energy and energy spread, or through to the next set of quadrupoles. After VESPER there are two further quadrupole triplets, a large drift space, followed by a quadrupole doublet, drift space and a final quadrupole installed in air next to the experimental test stand. A stainless steel vacuum beampipe runs through the beamline up to a beam exit window consisting of 0.1 mm thick mylar at 139 cm after QFD880. After this, the beam travels through air in the final quadrupole (QDD920) to the test stand area, where a 10 cm $\times$ 14 cm $\times$ 41 cm PMMA (polymethyl methacrylate) water tank, with 1 cm thick walls filled with liquid water was installed, as shown in Fig. 8, in addition to the film holders and a robot for moving the films into and out of the plane of the beam remotely.

The water tank was installed on a movable stage that could be moved into the plane of the beam (for the water irradiations) and out of the plane of the beam (for the air irradiations in which no tank was present). The water tank itself is made of 1 cm thick PMMA walls, which has a similar density to water, with a 5 cm diameter hole cut in the centre of the wall facing the beam. The hole is covered by a thin (0.1 mm) layer of Kapton, bypassing the need for the beam to be scattered by the tank walls before entering the water. The distance from the exit of the final quadrupole to the entrance of the water after the Kapton window entrance of the water tank was recorded as 12 cm.

The robot^41^ installed in the test stand area has a 3D range of movement and was used to place film holders and a YAG screen in front of the beam in the water tank or in air. The film holders were 12 cm long with 23 EBT-XD films cut to 35 mm $\times$ 35 mm, separated by 5 mm. The film holders and YAG screen were placed in line with the beam exit window and kapton window, ensuring the films were aligned with the beam and completely submerged in water or in air as required.

The YAG screen was used to measure the scaled dose and beam dimensions in real time, which minimised beam downtime during experiments. The YAG screen is made of a scintillating material, yttrium aluminum garnet activated by cerium (YAG:Ce), that is chemically resistant, making it suitable for use in X-ray and electron imaging systems. The robot film placement position resolution is 50 $\mu$m ($\sigma$). Using a camera mounted on the robotic arm in combination with the YAG screen on a specific holder, beam profiles and positions were recorded in water and air. Achieving focused VHEE beams at CLEAR was challenging due to the geometrically fixed quadrupole setup. It has been shown previously (and was observed within this experiment also) that the cross-sectional distribution on the YAG screen is typically in a good correspondence with that on the EBT-XD films over a wide range of the beam parameters^42^. The YAG screen gives a rapid readout though, in contrast to Gafchromic films, which require at least 12 hours of processing. For this reason, the YAG screen was used to scan the beam shape ($\sigma_x$ and $\sigma_y$) over a range of depths into the water phantom, for a range of quadrupole, beam energy and steering parameters. Once the optimal settings were found, the YAG screen was removed and the robot was used to place the GafChromic films into the path of the beam. The dose was obtained after processing the film.

### Monte Carlo simulations

**Figure 9:**
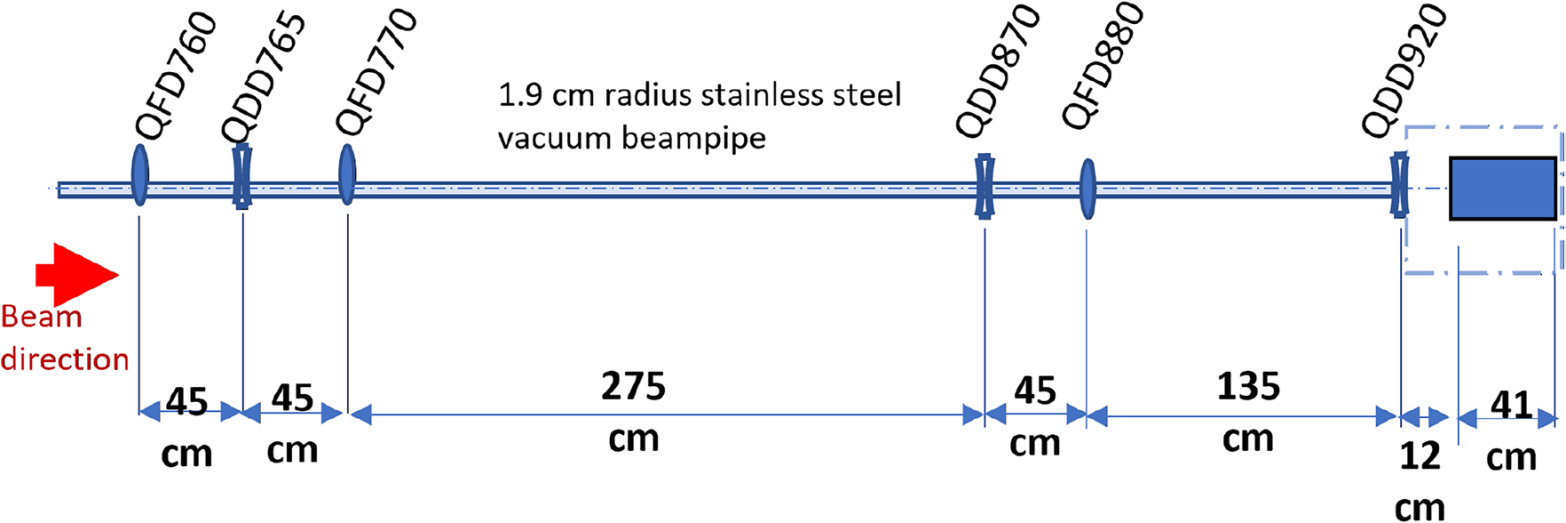
Schematic of the final six quadrupoles and water phantom used in each of the TOPAS Monte Carlo simulations of the CLEAR beam line. The air simulations have the same layout but with air instead of water in the phantom and 3.2 cm $\times$ 3.2 cm $\times$ 0.3 mm (dimensions of the films) boxes of water where the films were located, in place of the Gafchromic films.

Due to the many constituent parts along the CLEAR beamline, a simplified setup was modelled in TOPAS, consisting of the final six quadrupoles, stainless steel vacuum beam pipe and a water or air phantom at the positions shown in Fig. 9.

The quadrupole currents (A) for the quadrupoles used at CLEAR are converted into conventional quadrupole strengths (T/m) using Eq. (1)^43^:

1 $$\begin{aligned} 1 \text {A} = 0.057\, \text {T/m}. \end{aligned}$$

The quadrupole magnets were modelled as 40 cm $\times$ 40 cm $\times$ 22.6 cm (Height $\times$ Width $\times$ Length) boxes of air. The experimental beam energy of 201 MeV and 2 MeV energy spread, as well as the quadrupole strengths and positions, and the 3 mm thick, 1.9 cm radius stainless steel vacuum beampipe were modelled in TOPAS^27^ simulations. The angular divergence and initial beam $\sigma$ and bin size were chosen to be those giving the closest results to those recorded on the films for each irradiation (see “Data analysis” section). In Fig. 3, the quadrupole strengths shown in Supplementary Table 2 were used, with the final quadrupole strength changed also to 230 A and 250 A. For the air results shown in the Supplementary Materials, and the TOPAS simulations shown in Fig. 1, the quadrupole strengths shown in Supplementary Table 1 were used.

**Figure 10:**
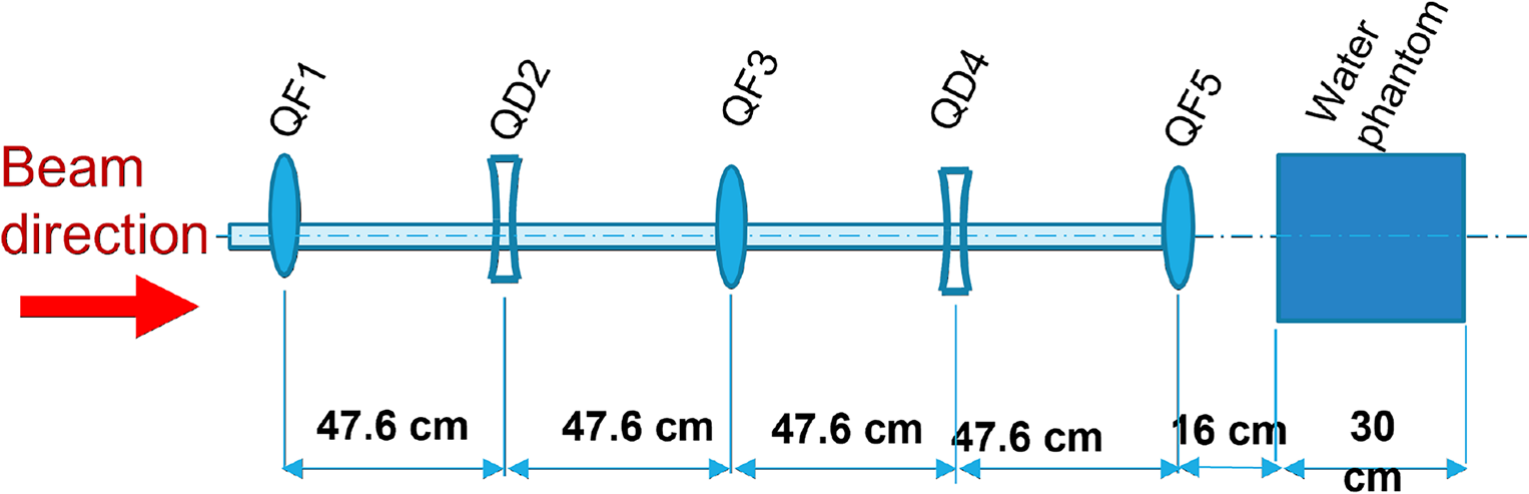
Schematic of the final five quadrupoles, stainless steel beampipe and water phantom used in the TOPAS simulations for the more compact beamline in Fig. 6. QF1, QF2 and QF3 are the three focusing quadrupoles, and QD1 and QD2 are the two defocussing quadrupoles.

For the optimised quadrupole setup shown in Figs. 6, 10 shows the layout used in the TOPAS Monte Carlo simulations.

Here, a 201 MeV VHEE beam with 2 MeV energy spread and 0.5 mm initial beam $\sigma$ was modelled, with 40 cm $\times$ 40 cm $\times$ 22.6 cm quadrupoles (consisting of air) and a 3 mm thick stainless steel vacuum beampipe of radius 1.9 cm (as well as 2.9, 3.9, 4.9, 5.9, 6.9 cm, 7.9 and 8.9 cm) each spaced 25 cm apart, and a 30 cm $\times$ 30 cm $\times$ 30 cm water phantom was placed 11 cm after the final quadrupole. The optimised quadrupole strengths used are shown in Supplementary Table 7 in the Supplementary Materials. This was a Monte Carlo simulation in which the position of each quadrupole can be arbitrarily precise. Note that the layout here has a spacing 47.6 cm—this is purely because of the dimensions of the quadrupole magnets being 22.6 cm, as is the case at CERN. If all of the quadrupoles were shifted by 1 mm it would not make a difference, and indeed the position with which the beam starts can change these values.

### Film calibrations

**Figure 11:**
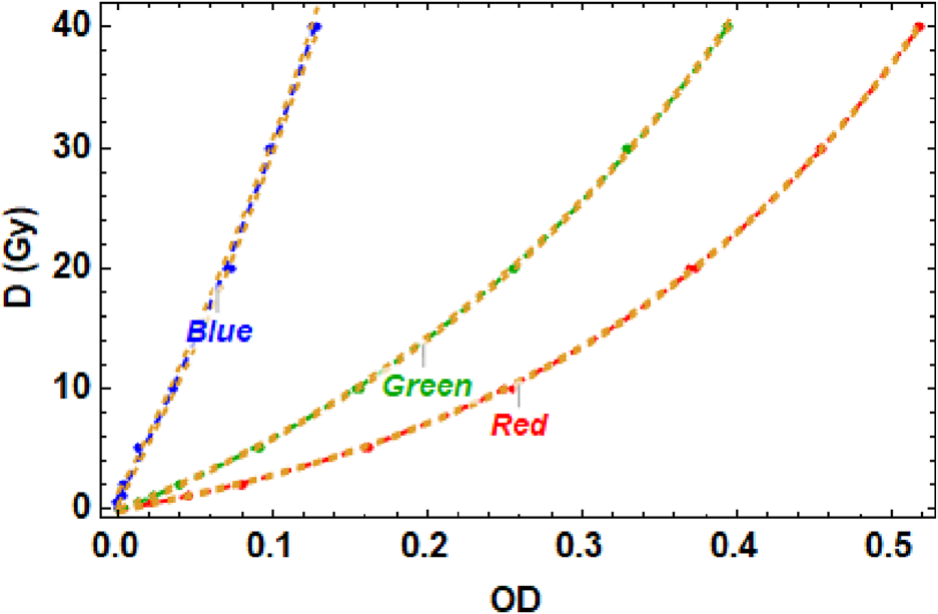
Calibration curve fits of dose, *D* as a function of Optical Density, *OD*, produced using 15 MeV electrons irradiating EBT-XD film at the Christie NHS hospital, for each of the three colour channels. All fits were based on Eq. (3). The yellow dashed lines indicate the 95% confidence levels.

All EBT-XD films used in the experiment were calibrated at the Christie NHS hospital in Manchester. Calibrations were performed using a 15 MeV clinical electron linac, as well as 6 MV photons from the linac and 150 MeV protons from a superconducting cyclotron. Here we only show the electron linac calibration. The dose range used was from 0.5 to 40 Gy, which is well within the quoted dynamic dose range of 0–60 Gy given by the manufacturers^44^. The films were irradiated and then scanned on the same high quality Epson scanner. The calibration films were scanned using two different scanners, once at the Christie hospital 24 hours after the irradiations took place, and again at CERN one week after the films were irradiated, which showed little variation in dose as expected (the main changes to optical density of the film take place within 12 hours after irradiation, see^45^.) This was done twice as the films from the experiment were scanned 12–24 hours after irradiation at CERN, and then again one week later on the Christie scanner, to check that the results were as expected on each scanner. The images were recorded with 300 dpi and in transmission mode to reduce further exposure to the films. The images were then converted into numerical 16-bit pixel data and the optical density was recorded for each of the three colour channels (RGB), calibrating for zero dose, by converting the pixel value to Optical Density (*OD*) using the following equation

2 $$\begin{aligned} OD = - \log _{10}(PV/PV_0), \end{aligned}$$

where *PV* is the pixel value of the exposed film, and $PV_0$ for the unexposed film. This was applied to each of the colour channels, red, green and blue. The equation to fit the dose (*D*) used was^46^:

3 $$\begin{aligned} D =a.b^{OD} + c \end{aligned}$$

where *a*, *b* and *c* are fit parameters found for each colour channel. The resultant calibration curves for the electron linac data are shown in Fig. 11.

Figure 11 shows the calibration doses obtained in 15 MeV electron mode using a clinical linac at the Christie hospital, with each recorded data point the mean of two recorded film doses for each monitor unit value across the range of 0–40 Gy. The fits, shown in the solid lines in the figure come from fitting the recorded dose and optical density data to Eq. (3). The resultant fit parameters and associated $\chi ^2$ values^47,48^ per degrees of freedom (or reduced $\chi ^2$ value^47^) are shown in Table 2.

**Table 2:** Calibration curve fit parameters and associated uncertainties for the curves shown in Fig. 11.

Colour channel	a (Gy)	b	c (Gy)	Adjusted $R^2$	Reduced $\chi ^2$
Red	5.05 ± 0.16	82.86 ± 4.60	− 5.08 ± 0.21	0.999917	1.18
Green	12.54 ± 0.39	18.78 ± 0.91	− 12.65 ± 0.43	0.999927	0.62
Blue	29.22 ± 1.88	66.17 ± 12.06	− 29.39 ± 1.95	0.999927	11.300

Note that the $\chi ^2$ value was considerably worse for the blue channel, and as a result was not used. This same analysis was also performed for proton and photon calibration films, with the resultant three particle modality calibrations obtained at the Christie hospital for 15 MeV electrons, 6 MV photons and 150 MeV protons.

**Figure 12:**
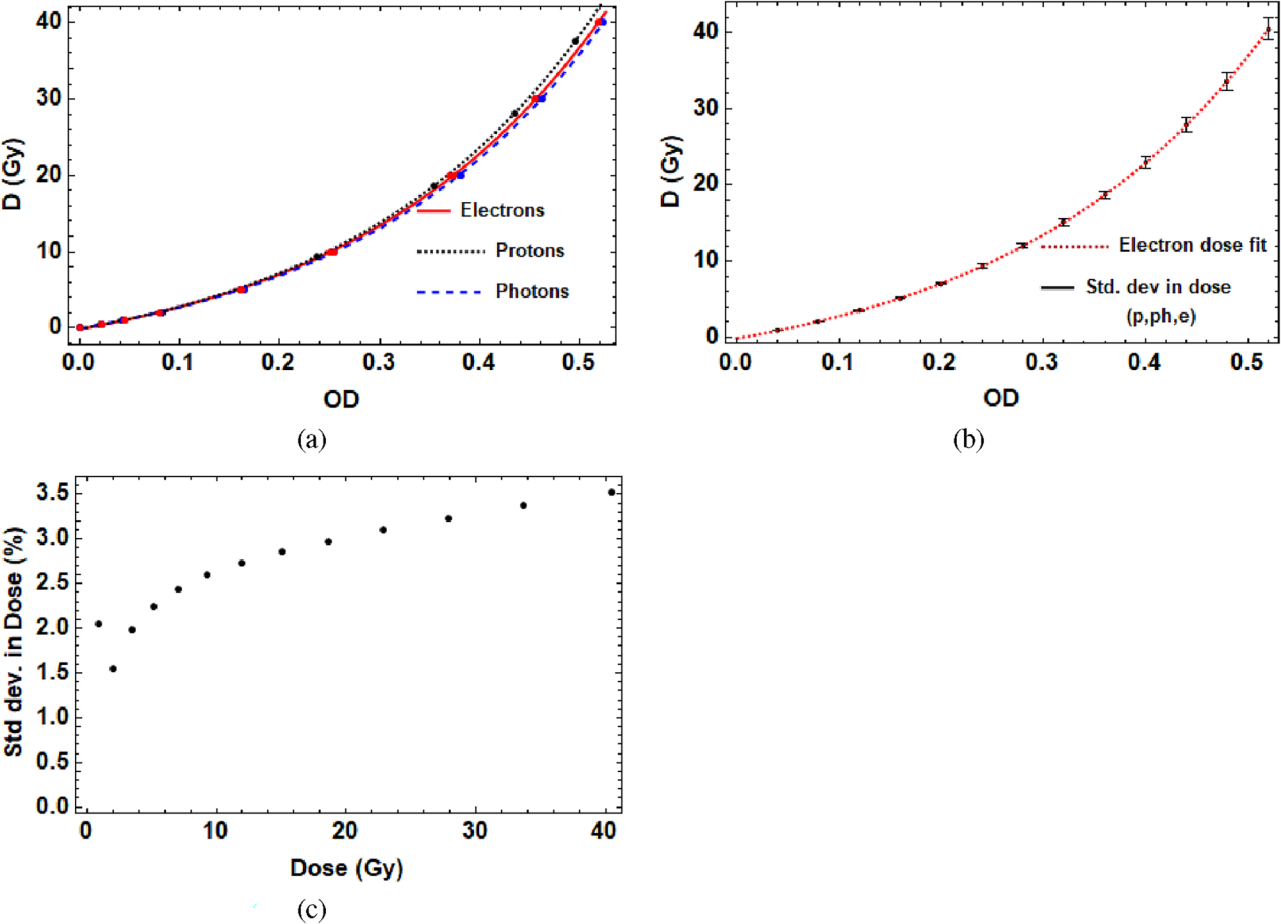
(**a**) Shows a comparison of the red channel calibration curves for EBT-XD film with 150 MeV protons ($\chi ^2$ = 0.21), 15 MeV electrons ($\chi ^2$ = 1.18) and 6 MV photons ($\chi ^2$ = 0.45). (**b**) Shows the standard deviation in dose across the three different particle modalities compared to the electron red channel fit, and (**c**) show the same information as (**b**) but with the standard deviation as a percentage of the electron fit result at that dose value.

Figure 12a shows the recorded dose data and fits for the red channel results (from Eq. (3)), and Fig. 12b shows the standard deviation in fits for a range of red channel doses for each of the particle modalities, with the fit for the electron calibration shown also, as this was the one chosen for the experiment. Figure 12c shows the percentage standard deviation for a range of the calculated electron dose values that were used in the experiment calibration. This shows that the maximum standard deviation in the calculated dose from the fits for each of the different particle modalities is 3.5%; this lends weight to the use of this method for the VHEE beams at CLEAR, for which no clinical reference beam for use in the calibrations currently exists.

### Determining dose

As prescribed by the manufacturers, the EBT-XD films were stored in a dark room after irradiations and 12 hours later were scanned on the same Epson scanner as the calibration films, also with 300 dpi. Due to the non-Gaussian nature of the dose recorded on the films for the strongly focused beams (due to collimation by the vacuum beampipe), the on-axis dose was determined by searching for the maximum dose in x and y and using a smoothing function (smoothing in steps of 10 pixels), to minimise any superficial marks on the films artificially increasing the dose.

**Figure 13:**
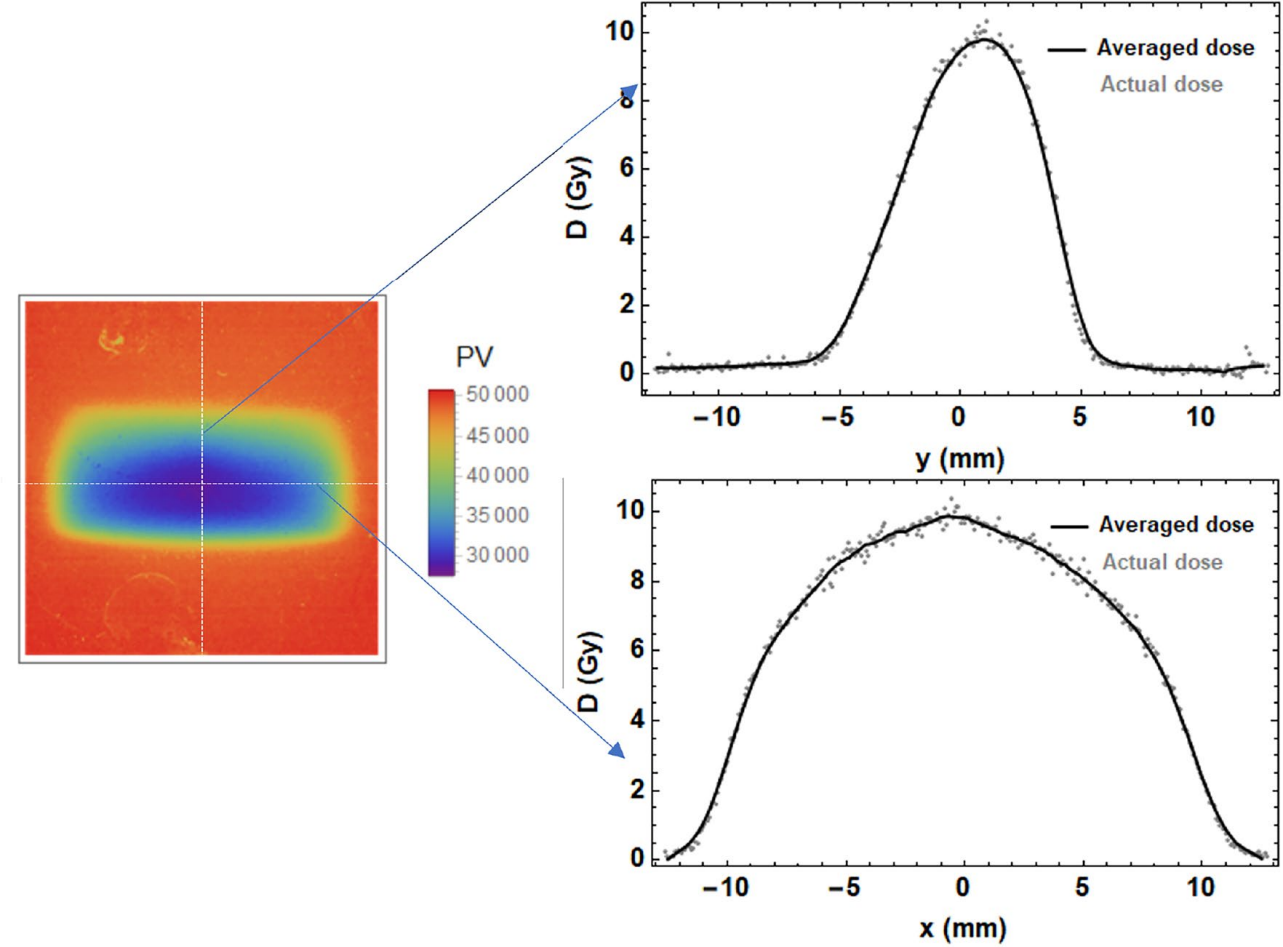
Shows one of the pixel value images generated using Mathematica for one of the irradiated films, as well as the profiles in the *x*- and *y*-planes at the position of maximum dose (located by finding in *x*- then searching in *y*- until no further change in either).

The pixel value plot image (which is used to find the optical density and then dose received by each of the pixels) of one of the films irradiated in the experiment is shown in Fig. 13, with the vertical and horizontal lines showing where the maximum dose is located in each plane shown. At the position of maximum dose, the vertical and horizontal lines were chosen as the location to find the FWHM in each plane, which was then used to compute the HWHMs. The dose was recorded as the average dose across each of the maximum lines in the *x*-plane and *y*-plane for the red channel dose, using the 15 MeV clinical electron beam calibration curve. This value was then compared with taking the average dose across a non-smoothed square of 10 $\times$ 10 pixels around the position of maximum dose, with the values found to agree within an average of 0.6%.

### Optimisations of focusing

The final six quadrupole magnets were used to produce the focused VHEE beams. The beam energy was measured at VESPER^40^ before each set of irradiations. A beam energy of 201 MeV was selected for each irradiation. In order to produce focused VHEE, the Twiss parameters at the start of the final six quadrupoles had to be accurately determined. This was done by performing quadrupole scans using a YAG screen to measure the beam size for a variety of quadrupole strengths at a chosen quadrupole within one of the quadrupole triplets, and using this to determine the Twiss parameters at the middle of the quadrupole, using the method described in the Supplementary Materials. These parameters were then used to optimise for focusing at a variety of depths into the phantom using optimisations performed using the Elegant code, as well as codes developed in house, described in the Supplementary Materials. Using the Twiss parameters and transfer matrix equations in this way inherently assumes vacuum conditions. For this reason, many optimisation parameters were obtained and then used with the YAG screen to determine whether focusing had occurred as predicted firstly in air and then if that was successful, in the water tank.

VHEE beams interact with the medium they traverse, and scattering interactions cause the beam size to increase with depth. This effect is shown clearly in Fig. 2, where without focusing the beam size increased from 1.5 mm in the x-plane and 1.7 mm in the y-plane at 1 cm into the water phantom, to 4.2 mm in both by 13 cm into the water phantom. This effect is proportionally stronger for smaller beams than larger beams, i.e. an increase in beam size of 4 mm in 15 cm of water has a much smaller effect on a beam size of initially 10 cm compared to 1 mm. Additionally, as focusing is a geometric effect, a focused beam arises from a larger beam size squeezing to a smaller beam size, with stronger focusing achievable when this difference is large over a short distance (i.e. sharpest angle possible between the size of the beam at the entrance and the size of the beam at the focal point). In a vacuum, the angular difference is the only concern when calculating how a beam will be affected by the focusing strengths used. In any other medium, the beam size is not only affected by the magnets, but also is scattered with depth due to interactions with the medium. For these reasons, a larger (1 cm or more) beam size is required at the entrance to result in a detectably focused beam at the target in water, and the focal point predicted by vacuum accelerator codes will be further into the water than the one actually observed.

The beam size at CLEAR without interference is very small (< 2 mm). For this reason, in this experiment the emittance (corresponding to the beam size) was artificially increased by inserting a screen at position 810 (see Fig. 7), up to > 3 cm from < 2 mm in the absence of focusing, allowing for sharper focused VHEE to be achieved than was possible without doing so.

In this experiment, highly focused beams were achieved, with the focal point shifted closer to the entrance of the phantom than predicted by the codes, due to scattering in air and water, and collimation of the beam due to the relatively small beampipe aperture (3.8 cm diameter). Due to the positions of the quadrupoles (see Fig. 9), it was not possible to focus in both planes; as such focusing was concentrated in one plane, with a beam that was gently diverging in the other plane.

### Data analysis

All TOPAS simulations and Gafchromic films were analysed using code written in the Mathematica^49^ language. Figure 1 was produced using parameter files written in TOPAS with the layout shown in Fig. 9 for the quadrupole strengths shown in Supplementary Table 1, the dose in the central 0.1 cm $\times$ 0.1 cm $\times$ 41 cm region for each case is shown. For Fig. 2, the he dose in the central 0.3 cm $\times$ 0.3 cm $\times$ 41 cm region is shown for the focused VHEE dose distribution. For Fig. 3, the dose in the central 0.3 cm $\times$ 0.3 cm $\times$  41 cm region is shown for each case. For Fig. 4, the dose in the central 0.3 cm $\times$ 0.3 cm $\times$ 41 cm region is shown.

In Fig. 6, the dose in the central 0.3 cm $\times$ 0.3 cm $\times$ 30 cm was recorded for the quadrupole settings shown in Supplementary Table 3 for a final quadrupole strength of 240 A.

For the experimental data shown in Figs. 2, 3, and 4, the dose was recorded on Gafchromic films held in place by the film holders and processed using the method shown in the “Determining dose” section. The errors on the experimental data in Figs. 2, 3, and 4 were determined using the method shown in the “Error analysis” section.

In Fig. 3b, the data shown shows the position of the maximum dose in the TOPAS simulations shown in Fig. 3a (found using code written in the Mathematica language). The error bars here are the discrepancy between the Monte Carlo simulation position and the recorded position on maximum dose on the films (which were each separated by 5 mm). A linear fit was used to fit the data set, and the goodness of fit was evaluated using the adjusted $R^2$, where $R^2$ is the coefficient of multiple determination, calculated by the Mathematica code. Details about how the adjusted $R^2$ is calculated can be found in e.g.^50^. Essentially it is a measure of how well the data fits the model prediction, with the adjusted $R^2$ also taking into account the number of degrees of freedom used, adding a penalty for artificially ’good’ fits due to simply adding further parameters to the model that may not accurately describe the actual behaviour, as is the case for $R^2$ when it is not the adjusted value. An ideal $R^2$ value is 1, with the number of decimal points to which it rounds to 1 the best marker for whether the fit is ’good’ or not. The Mathematica calculated adjusted $R^2$ is also used to assess the goodness of fit of the calibration data shown in Fig. 11, as well as for Fig. 11 a $\chi ^2$ value, which uses a least squares analysis to consider the difference between the observed and expected (i.e. model) values for each of the given data points. Details about the calculation of $\chi ^2$ can be found for instance in^47,48^.

For Figs. 11 and 12, the method for obtaining and analysing the data is described in the “Film calibrations” and “Determining dose” sections.

### Error analysis

There were multiple sources of experimental uncertainty. For the dose values shown from the experiment, the uncertainties were determined to be due to the discrepancy in absolute dose between the green and red colour channels (an average of 4%), the discrepancy in dose response of the films due to the difference in modality of calibration used (3.5% at the highest dose value used, less for lower doses) and the difference between taking the smoothed pixel value for the maximum dose compared to averaging over a small rectangle of 10 $\times$ 10 pixels (an average of 0.6%), added in quadrature, giving an approximate uncertainty of 5%. Note that this is higher than the quoted 2% given by the manufacturers^45^.

The uncertainty in the HWHMs is given by the average percentage difference (2% in x and 4% in y for the normal polarity quadrupoles, and the reverse for when they were inverted) when calculating the HWHMs using the different calibration modalities. For the uncertainties in the position of maximum dose shown in Fig. 3, the uncertainty is given by the difference in position of maximum dose recorded on the film compared to the TOPAS Monte Carlo simulation result.

### Supplementary Information

Supplementary Information.

## Data Availability

The datasets generated during and/or analysed during the current study are available from the corresponding author on reasonable request.
