# Supplementary material for: CERN-based experiments and Monte-Carlo studies on focused dose delivery with very high energy electron (VHEE) beams for radiotherapy applications
Source: Sci Rep. 2024 May 15;14:11120. doi: 10.1038/s41598-024-60997-5 (PMC11096185; doi:10.1038/s41598-024-60997-5)
Supplement: Supplementary file 1 — Supplementary Information. [file 41598_2024_60997_MOESM1_ESM.pdf]

CERN-based Experiments and Monte-Carlo Studies on Focused and SOEP Dose Delivery with Very High Energy Electron (VHEE) Beams for Radiotherapy Applications - Supplementary Materials

0.1 Determining Twiss Parameters

There several ways of determining the Twiss parameters (α, β, γ) and transverse emittance at a particular point along the beamline: one method is through the use of 'quadrupole scans'[1], and another is through the multiple profile monitor method[2]. For the Twiss parameters stated the in the main manuscript, the quadrupole scan method was used to determine them, which is the most commonly used method.

0.1.1 Quadrupole Scan

Quadrupole scans are used to determine the transverse emittance, ε and Twiss parameters (α, β and γ), by making use of one quadrupole, a drift space and an imaging tool capable of determining the beam size[1]. Along the path of the accelerator, particles are contained within an ellipse in phase space, which can be described mathematically by the beam matrix, σ_b,

$$\sigma_b = \begin{pmatrix} \sigma_{11} & \sigma_{12} \\ \sigma_{21} & \sigma_{22} \end{pmatrix} = \varepsilon \begin{pmatrix} \beta & -\alpha \\ -\alpha & \gamma \end{pmatrix}. \tag{1}$$

where σ_{11} is the rms (root mean squared) beam size, σ_{22} is the rms divergence, and $\sigma_{12} = \sigma_{21}$ are the cross-terms. The geometric emittance of the beam can be found from

$$\varepsilon = \sqrt{\det(\sigma_b)}, \tag{2}$$

where $\det(\sigma_b)$ is the determinant of σ_b. For a beam that starts at the centre of a quadrupole, with corresponding beam matrix (σ_q), after traversing the rest of the quadrupole and drift space to the imaging position (motion described by the transfer matrix, M), the final beam matrix at the end of the drift space, σ_f can be found via

$$\sigma_f = M\sigma_q M^T. \tag{3}$$

For simplicity and to a good approximation of beam transport, the transfer matrix of a thin lens focusing quadrupole of focal length f, and a drift space of length d, from the centre of the thin quadrupole to the imaging position, can be written

$$M = \begin{pmatrix} 1 & d \\ 0 & 1 \end{pmatrix} \begin{pmatrix} 1 & 0 \\ -1/f & 1 \end{pmatrix} = \begin{pmatrix} 1-d/f & d \\ -1/f & 1 \end{pmatrix}. \tag{4}$$

Noting that $f = 1/(Kl)$ (K is the quadrupole field strength, and l is the length), the first element of the beam matrix at the imaging position, $\sigma_{f,11}$, which corresponds to the measured rms beam size on the imaging screen, can be written

$$\sigma_{f,11} = (1-dKl)^2\sigma_{q,11} + 2d(1-dKl)\sigma_{q,12} + d^2\sigma_{q,22}. \tag{5}$$

This can be written as a parabolic equation in terms of focusing strength, K,

$$\sigma_{f,11}(K) = d^2\sigma_{q,11}(Kl)^2 + (-2d - 2d^2\sigma_{q,12})(Kl) + (d^2\sigma_{q,22} + \sigma_{q,11} + 2d\sigma_{q,12}). \tag{6}$$

In a quadrupole scan, for the chosen quadrupole, the smallest beam size (corresponding to the focal point) must be found, and the quadrupole scanned in terms of K around this position, with the measured beam size corresponding to $\sigma_{f,11}(K)$ measured. By equating $\sigma_{f,11}(K)$ as a function of K,

$$\sigma_{f,11}(K) = aK^2 + bK + c, \tag{7}$$

to equation (6), the fit parameters can then be found, and used to determine the quadrupole beam matrix elements,$\sigma_{q,ij}$, i.e. the conditions at the centre of the quadrupole, in equation (3).
This can finally be related back to the initial Twiss parameters at the centre of the quadrupole via equation (1) to give

$$\varepsilon = \sqrt{\sigma_{q,11}\sigma_{q,22} - \sigma_{q,12}^2} \tag{8}$$

$$\beta_q = \frac{\sigma_{q,11}}{\varepsilon} \tag{9}$$

$$\alpha_q = -\frac{\sigma_{q,12}}{\varepsilon} \tag{10}$$

$$\gamma_q = \frac{\sigma_{q,22}}{\varepsilon}. \tag{11}$$

Note that as quadrupoles focus in one plane and de-focus in the other, the horizontal emittance and Twiss parameters must be found using a focusing quadrupole, whilst the vertical emittance and Twiss parameters must be found using a defocusing quadrupole. This will result in Twiss parameters recorded at different locations; for them to be measured at the same point they must be back-tracked to the same starting point. This was the method used to determine the Twiss parameters in the main manuscript, and the imaging method used to determine the rms beam size at the imaging location was a YAG screen, described in the Methods section.

0.1.2 Multiple Profile Monitor Method

Another method for determining the beam emittance and Twiss parameters is through the multiple profile monitor method[2]. This involves using a drift space of three different lengths (l_a, l_b, l_c) and measuring the rms beam size at each length (w_a, w_b, w_c), to determine the Twiss parameters at the start of the drift space. This is a similar method to that described in the quadrupole scan section, but this time the transfer matrix, M_{l_n}, consists only of the drift space of length l_n,

$$M_{l_n} = \begin{pmatrix} 1 & l_n \\ 0 & 1 \end{pmatrix}. \tag{12}$$

For the first drift space, with $l_n = l_a$, equation (3) can be written instead as

$$\sigma_a = M_{l_a} \sigma_i M_{l_a}^T \tag{13}$$

with σ_i that at the beginning of the drift space, and σ_a the beam matrix at the end of the drift space. For the three different drift space lengths, l_a, l_b and l_c, there will be three corresponding versions of equation (13). The first element of the beam matrix in equation (13), (i.e. the rms of the beam size, w_a^2 at position l_a), $\sigma_{a,11} = w_a^2$ is therefore

$$w_a^2 = \sigma_{i,11} - 2 l_a \sigma_{i,12} + l_a^2 \sigma_{i,22}, \tag{14}$$

with similar equations for w_b and w_c. Combining the three into a matrix formulation gives

$$\begin{pmatrix} w_a^2 \\ w_b^2 \\ w_c^2 \end{pmatrix} = \begin{pmatrix} 1 & -2l_a & l_a^2 \\ 1 & -2l_b & l_b^2 \\ 1 & -2l_c & l_c^2 \end{pmatrix} \begin{pmatrix} \sigma_{i,11} \\ \sigma_{i,12} \\ \sigma_{i,22} \end{pmatrix}. \tag{15}$$

Then, recalling that σ_b can be written in terms of the Twiss parameters, α, β, γ using equation (1), we finally arrive at

$$\begin{pmatrix} w_a^2 \\ w_b^2 \\ w_c^2 \end{pmatrix} = \begin{pmatrix} 1 & -2l_a & l_a^2 \\ 1 & -2l_b & l_b^2 \\ 1 & -2l_c & l_c^2 \end{pmatrix} \begin{pmatrix} \varepsilon\beta \\ -\varepsilon\alpha \\ \varepsilon\gamma \end{pmatrix}. \tag{16}$$

Taking the inverse of the l matrix allows us to find the original Twiss parameters at the start of the drift space, after w_a, w_b, w_c have been measured using the YAG screen or a similar device. This method was particularly useful as a modelling tool for Monte Carlo simulations of the dose during the experiment, in which the beam size for a particular set of quadrupole magnets was measured at various points in air, and used to determine the Twiss parameters at the end of the final quadrupole, which were then used as input into a much faster simulation of just the water tank to see whether focusing was achieved in water, compared to having to model the whole beamline, or to require film processing time.

0.2 Focusing Optimisations

The focusing optimisations use the fact that at the focal point, $\alpha = 0$, and β should be as small as possible (corresponding to a small beam size at that location). Knowing the Twiss parameters before the final six quadrupoles means that the following quadrupole strengths can be used as parameters to produce such conditions at the focal point through optimisations, i.e. using

$$\varepsilon \begin{pmatrix} \beta_f & -\alpha_f \\ -\alpha_f & \gamma_f \end{pmatrix} = M \varepsilon \begin{pmatrix} \beta_0 & -\alpha_0 \\ -\alpha_0 & \gamma_0 \end{pmatrix} M^T \tag{17}$$

where α_0, β_0 and γ_0 are the Twiss parameters at the entrance of the first quadrupole (in the final six to be optimised), and α_f, β_f and γ_f are the Twiss parameters at the focal point. ε is the beam emittance. M is the transfer matrix consisting of the 6 quadrupole magnets and corresponding drift spaces,

$$M = M_{l_6} M_{D_3} M_{l_5} M_{F_3} M_{l_4} M_{D_2} M_{l_3} M_{F_2} M_{l_2} M_{D_1} M_{l_1} M_{F_1} \tag{18}$$

where M_{F_i} are the transfer matrices for the focusing quadrupoles of length L_i (m) and focusing strength K_i (m^{-2}),

$$M_{F_i} = \begin{pmatrix} \cos\Theta_i & \frac{1}{\sqrt{|K_i|}} \sin\Theta_i \\ -\sqrt{|K_i|} \sin\Theta_i & \cos\Theta_i \end{pmatrix} \tag{19}$$

where $\Theta_i = \sqrt{|K_i|} L_i$. Similarly, M_{D_i} are the transfer matrices for the defocusing quadrupoles of length L_i (m) and focusing strength $-|K_i|$ (m^{-2}),

$$M_{D_i} = \begin{pmatrix} \cosh\Theta_i & \frac{1}{\sqrt{|K_i|}} \sinh\Theta_i \\ \sqrt{|K_i|} \sinh\Theta_i & \cosh\Theta_i \end{pmatrix}. \tag{20}$$

and finally M_{l_i} are the transfer matrices for the drift spaces of length l_i (m) as defined in Equation (12).

0.3 Experimental Quadrupole and Beam Settings

Supplementary Table 1. Quadrupole currents for dose distribution shown in Figure 1 in main manuscript.

Quadrupole	Current (A)
QF760	68.5
QD765	106
QF770	48
QD870	10
QF880	69.5
QF910	195

Supplementary Table 2. Quadrupole currents for dose distributions shown in Figure 2 and Figure 5 in main manuscript.

Quadrupole	Current (A)
QF760	68.5
QD765	106
QF770	48
QD870	131
QF880	104
QF910	240

Supplementary Table 3. Reconstructed Twiss parameters determined using quadrupole scans for the beam parameters at CLEAR at the first of the final 6 quadrupoles. Used for the input parameters in the focusing optimisations, with the beam emittance ε artificially increased using a YAG screen, as described in the Methods section in the main manuscript.

Parameter	
α_x	-13.28
β_x	69.44 m
$\varepsilon_{x,norm}$	984.9 mm mrad
α_y	-17.53
β_y	72.27 m
$\varepsilon_{y,norm}$	881.2 mm mrad

Supplementary Table 4. Quadrupole Strengths for dose distributions shown in Figure 8.

Quadrupole	Strength (T/m)
QF1	20.6
QD2	-13.1
QF3	7.7
QD4	-13.4
QF5	15.3

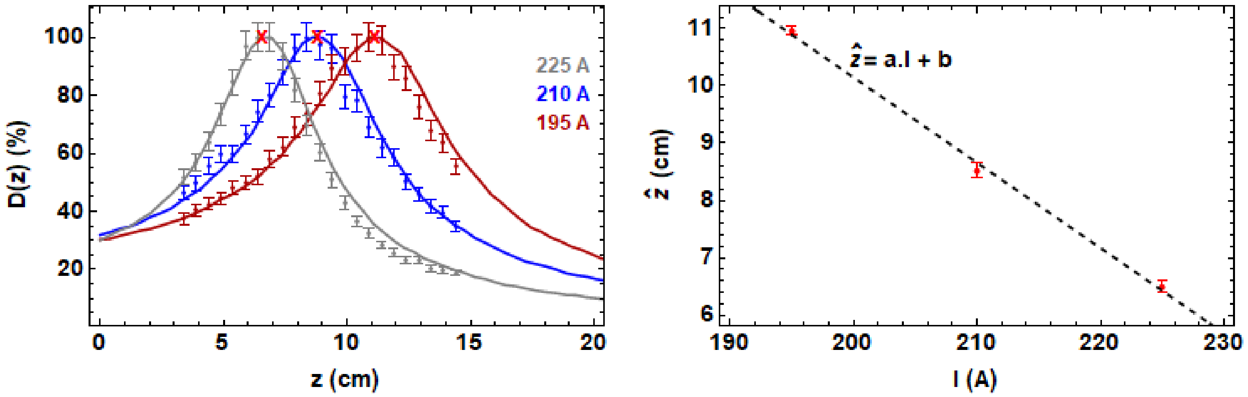

(a) (b)

Supplementary Figure 1. (a) Percentage on-axis dose due to changing the final quadrupole current in air. Points are experimental data, and lines are Monte Carlo simulations. (b) A linear fit of peak dose position to final quadrupole current, a=-0.15±0.01 cm/A, b=39.9±1.64 cm and R^2=0.9996. The red crosses indicates the peaks in both a) and b).

0.4 Focusing in Air at the CLEAR Facility

The quadrupole strengths used to produce Supplementary Figure 1 are shown in Supplementary Table 1. The conversion from quadrupole current (A) to conventional quadrupole strengths (T/m) is shown in equation 1 in the Methods section of the main manuscript.

References

1. Green, A. T. & Shin, Y. M. Implementation of quadrupole-scan emittance measurement at fermilab's advanced superconducting test accelerator (asta), DOI: 10.18429/JACoW-IPAC2015-MOPMA052.

2. McDonald, K. T. & Russell, D. P. Methods of emittance measurement. In Month, M. & Turner, S. (eds.) *Frontiers of Particle Beams; Observation, Diagnosis and Correction*, 122–132 (Springer Berlin Heidelberg, Berlin, Heidelberg, 1989).
